# The Optoelectronic Properties of p-Type Cr-Deficient Cu[Cr_0.95−x_Mg_0.05_]O_2_ Films Deposited by Reactive Magnetron Sputtering

**DOI:** 10.3390/ma13102376

**Published:** 2020-05-21

**Authors:** Song-Sheng Lin, Qian Shi, Ming-Jiang Dai, Kun-Lun Wang, Sheng-Chi Chen, Tsung-Yen Kuo, Dian-Guang Liu, Shu-Mei Song, Hui Sun

**Affiliations:** 1 The Key Lab of Guangdong for Modern Surface Engineering Technology, National Engineering Laboratory for Modern Materials Surface Engineering Technology, Guangdong Institute of New Materials, Guangzhou 510651, China; lss7698@126.com (S.-S.L.); qianzixlf@163.com (Q.S.); daimingjiang@tsinghua.org.cn (M.-J.D.); huisun@sdu.edu.cn (H.S.); 2 Shandong Key Laboratory of Optical Astronomy and Solar-Terrestrial Environment, School of Space Science and Physics, Shandong University, Weihai 264209, China; wkl@sdu.edu.cn (K.-L.W.); songshumei@sdu.edu.cn (S.-M.S.); 3 Department of Materials Engineering and Center for Plasma and Thin Film Technologies, Ming Chi University of Technology, Taipei 243, Taiwan; 4 College of Engineering, Chang Gung University, Taoyuan 333, Taiwan; 5 Institute of Materials Science and Engineering, National Taiwan University, Taipei 106, Taiwan; d99527016@ntu.edu.tw; 6 School of Materials Science and Engineering, Southwest Jiaotong University, Chengdu 610031, China; dianguang@swjtu.edu.cn

**Keywords:** p-type conductivity, Cr-deficient CuCrO_2_, reactive magnetron sputtering, optoelectronic property

## Abstract

CuCrO_2_ is one of the most promising p-type transparent conductive oxide (TCO) materials. Its electrical properties can be considerably improved by Mg doping. In this work, Cr-deficient CuCrO_2_ thin films were deposited by reactive magnetron sputtering based on 5 at.% Mg doping. The influence of Cr deficiency on the film’s optoelectronic properties was investigated. As the film’s composition varied, CuO impurity phases appeared in the film. The mixed valency of Cu^+^/Cu^2+^ led to an enhancement of the hybridization between the Cu*3d* and O*2p* orbitals, which further reduced the localization of the holes by oxygen. As a result, the carrier concentration significantly improved. However, since the impurity phase of CuO introduced more grain boundaries in Cu[Cr_0.95−x_Mg_0.05_]O_2_, impeding the transport of the carrier and incident light in the film, the carrier mobility and the film’s transmittance reduced accordingly. In this work, the optimal optoelectronic performance is realized where the film’s composition is Cu[Cr_0.78_Mg_0.05_]O_2_. Its Haacke’s figure of merit is about 1.23 × 10^−7^ Ω^−1^.

## 1. Introduction

Transparent conductive oxides (TCOs) combine good conductivity and ideal transmittance and can be used in various domains [1,2,3]. However, most commercially used TCOs are n-type conductivity in which the majority carriers are electrons [4]. In fact, most oxides are intrinsic n-type conductive semiconductors. Due to the self-compensation effect, the p-type conductivity obtained by doping in n-type TCOs is unstable [5,6]. Meanwhile, for the limited intrinsic p-type oxides, since the valence band maximum is mainly occupied by O*2p* orbital, the strong electron negativity of oxygen ions localizes holes around them, resulting in a decrease in the carrier concentration and carrier mobility [7,8]. Thus, their p-type conductivity is generally poor. For the above reasons, p-type TCOs with ideal optoelectronic performance are difficult to fabricate [9,10,11]. p-type TCOs are an essential part in the building of fully transparent electronic devices and also play an imperative role as hole transport layers (HTLs) in novel perovskite solar cells and hole injection layers in organic light-emitting diode (OLED) displays [12,13,14]. In this context, lots of effort has been paid to developing p-type TCOs. Copper oxides are considered to be promising p-type TCOs [15,16]. The hybridization between Cu*3d* and O*2p* orbits effectively reduces the localization of the holes by oxygen ions, which is beneficial to improving the p-type conductivity of TCOs [17]. In particular, in 2001, Tate et al. reported that the conductivity of delafossite CuCrO_2_ film doped with Mg can reach 220 S·cm^−1^, which makes p-type TCOs present optimistic application prospects [18]. However, the film’s corresponding transmittance in the visible region is around just 30%.

Delafossite oxides have been widely studied in the past few decades. The conductivity of undoped delafossite materials is not very good [19]. However, their carrier concentration can be effectively enhanced using the doping method, thereby improving their electrical property [20,21]. Fang et al. analyzed the extrinsic defects in CuCrO_2_ using the first-principles methods and found that for all the acceptor-type extrinsic defects, substituting Mg for Cr is the most prominent doping acceptor with relatively shallow transition energy levels in CuCrO_2_ [22]. Its conductive mechanism can be depicted by the following equation:

(1) $${\left(Cr_{Cr}\right)}^{\times }+Mg\to {\left(Mg_{Cr}\right)}^{\prime }+\mathrm{Cr}+h^{+}$$

where (*Cr_Cr_*)^×^ represents the Cr in the original lattice sites, (*Mg_Cr_*)’ represents the Mg^2+^ in Cr^3+^ site and *h^+^* is the positive hole. To date, the highest p-type conductivity of about 278 S·cm^−1^ has been obtained in Mg and N co-doped CuCrO_2_ film deposited by radiofrequency (RF) sputtering, where the Cu vacancies (*V_Cu_*) as well as the substitution of Cr by Mg (*Mg_Cr_*) and O by N (*N_O_*) are considered as the intrinsic acceptor and extrinsic acceptor, respectively [23]. All of them contribute to the carrier concentration. Generally, a Cu-deficient condition is considered easier to generate Cu vacancy and is in favor of the film’s p-type conductivity. However, Chen et al. reported that Cr-deficient conditions can realize higher carrier concentrations [24]. This is because where Cr is deficient, Cu atoms in the structure might occupy Cr sites to form anti-site (*Cu_Cr_*) defects, leading to increased hole concentration. This process can be described as the following:

(2) $${\left(Cu_{Cu}\right)}^{\times }+{\left(Cr_{Cr}\right)}^{\times }\to {\left(V_{Cu}\right)}^{\prime }+{\left(Cu_{Cr}\right)}^{\prime \prime }+3h^{•}$$

where (*Cu_Cu_*)^×^ and (*Cr_Cr_*)^×^ represent the Cu and Cr in their original lattice sites, (*V_Cu_*) is the Cu vacancy, (*Cu_Cr_*) is the Cu in Cr site and *h^•^* is the compensated hole. As a result, the film’s p-type conductivity is improved. Other works also support this conclusion [25]. So far, there is no report about reinforcement of the optoelectronic properties of CuCrO_2_ thin films by Mg doping and the introduction of Cr deficiency at the same time. Thus, in this work, Mg doped Cr-deficient CuCrO_2_ films were deposited in order to optimize the p-type conductivity of CuCrO_2_. The influence of Cr content on the film’s optoelectronic properties is discussed in detail.

## 2. Materials and Methods

Mg doped Cr-deficient CuCrO_2_ films with a thickness of about 350 nm were deposited by reactive magnetron sputtering with direct current (DC) power supply at room temperature. This method allows perfect control of the film’s composition and possesses a higher deposition rate compared to RF sputtering. p-type silicon (100) wafer and fused quartz were used as substrates. Before the deposition, the substrates were ultrasonically cleaned successively using ultrapure water, acetone and alcohol for 15 min, initially. After cleaning, the residual alcohol on the substrates was blow-dried using a high-purity nitrogen gas. Pure copper, chromium and magnesium targets (99.99% in purity) each with a 50.8 mm diameter and 3 mm thickness were powered by pulsed DC supplies. Before the deposition, the background of the reactive chamber was pre-pumped to 10^−5^ Pa. Then, a gas mixture of Ar + O_2_ was introduced into the chamber. The flow rate was fixed at 90 and 10 sccm, with the working pressure fixed at 0.9 Pa. During the deposition, the pulsed frequency of each power supply was fixed at 50 kHz, while the pulse off-time was maintained at 5 μs. The discharge current applied on the Cu, Cr, Mg targets was varied as 0.14–0.15 A, 0.90–0.98 A, 0.28–0.32 A (the corresponding sputtering powers were 29–32 W, 241–257 W, 27–32 W) in order to deposit the films with various compositions. The deposition rate was about 5.5–6.0 nm/min. Herein, all the Cr-deficient films were Cu stoichiometric with 5 at.% Mg doping. Meanwhile, Cr content varied from 0.95 to 0.58. Thus, the chemical formulas of all films can be written as Cu[Cr_0.95−x_Mg_0.05_]O_2_, where the x values are 0.00, 0.09, 0.17, 0.23, and 0.37, respectively. Finally, all the films were annealed at 1023 K in a vacuum for 30 min in order to obtain a well crystallized delafossite structure.

The film’s thickness was determined by a surface profilometer (Ambios Technology Company, Santa Cruz, NM, USA). The film’s composition was confirmed by energy-dispersive spectroscopy (EDS, Nova Nano SEM 450, Hillsboro, OR, USA). The phase structures were analyzed by an X-ray diffractometer (XRD, Bruker D8 ADVANCE, Karlsruhe, Germany). Hall effect analysis with van der Pauw’s configuration (Keithley-4200 SCS, Beaverton, OR, USA) was used to investigate the film’s electrical properties under room temperature. The geometrical size of the rectangular sample is 1 cm × 1 cm. Finally, the film’s optical properties in the visible region and near infrared region were characterized by a UV-Vis spectrophotometer (Shimadzu UV-3600, Kyoto, Japan).

## 3. Results and Discussion

**Figure 1:**
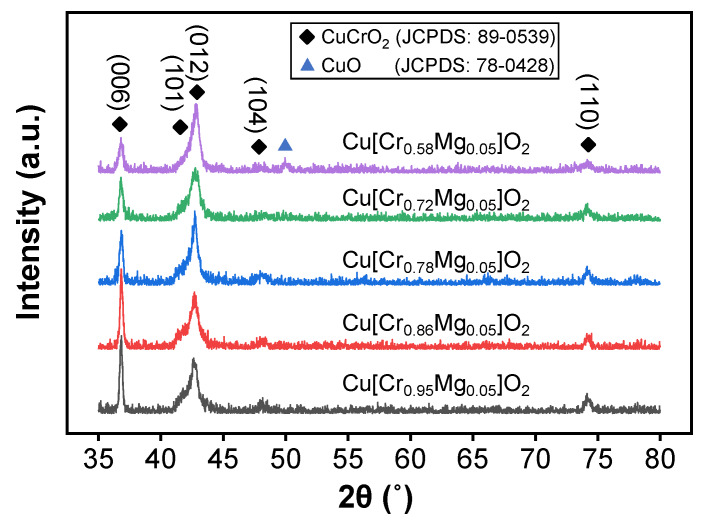
The XRD patterns of Cr-deficient Cu[Cr_0.95−x_Mg_0.05_]O_2_ films with various Cr content.

The XRD patterns of Cr-deficient Cu[Cr_0.95−x_Mg_0.05_]O_2_ films are compared in Figure 1. The diffraction peaks at 36.8°, 41.7°, 42.8°, 47.9°, and 74.3° corresponded to the (006), (101), (012), (104), and (110) orientations of 3R-CuCrO_2_ delafossite structure (JCPDS: 89-0539), respectively. With the decrease in Cr content, the preferred orientation changed from (006) to (012) plan. It is pointed out that the growth of CuCrO_2_ along the c-axis was beneficial to improving its conductivity [26]; however, in 3R-CuCrO_2_, the (012) plan owned lower surface energy. Therefore, the Cr-deficient condition in the current work is more conducive to the growth of CuCrO_2_ within thermodynamic equilibrium conditions. Moreover, as Cr content decreased to 0.58, a CuO phase emerged. Daou et al. reported that the substitution of Cr^3+^ by Mg^2+^ in CuCrO_2_ could lead to the formation of CuO (as Mg content above 0.04) [27]. As a result, a mixed valency of Cu^+^/Cu^2+^ was induced by the Mg^2+^ substitution on the Cr^3+^ site. In fact, increasing Cr deficiency by either reducing the Cr content or replacing Cr with Mg caused a conversion of monovalent Cu to divalent Cu. This was corroborated by XANES (X-ray absorption near-edge structure) spectra theoretical calculation [28]. This behavior changed the densities of Cu*3d*, Cu*3d*–O*2p* and O*2p* states at or near the valence-band maximum or the Fermi level, which further affected the film’s p-type conductivity.

**Figure 2:**
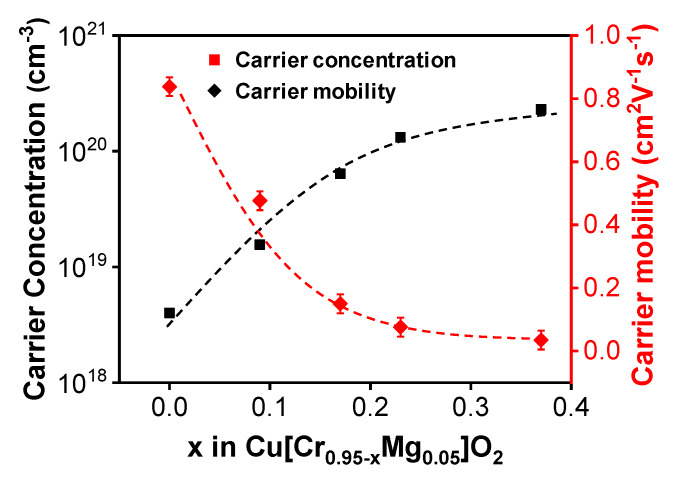
The carrier mobility and carrier concentration of Cr-deficient Cu[Cr_0.95−x_Mg_0.05_]O_2_ films as a function of Cr content.

The films’ electrical properties were analyzed by Hall measurement. All the films with various compositions presented p-type conductivity. The variation of the carrier concentration and carrier mobility as a function of Cr content is shown in Figure 2. It can be seen that as Cr deficiency increased, the carrier concentration increased. As mentioned above, after the substitution of Cr^3+^ by Mg^2+^, monovalent Cu converted into divalent Cu. This resulted in an enhancement of the hybridization between Cu*3d* and O*2p* orbitals [25]. As a result, the localization effect of the holes by oxygen was diminished, and the hole carriers were thereby released around the Cu sites. Chen et al. reported that under Cr-deficient condition, Cu atoms can occupy Cr sites and form (*Cu_Cr_*) anti-site defects [24]. During this process, Cu vacancies as well as three holes are generated, as shown in Equation (2) mentioned before. As a consequence, the carrier concentration is significantly enhanced from 4.0 × 10^18^ cm^−3^ to 2.3 × 10^20^ cm^−3^ as Cr content decreases from 0.95 to 0.58. It can be seen that Cu[Cr_0.95−x_Mg_0.05_]O_2_ becomes degenerate as the carrier concentration increases [29].

As for the carrier mobility, its variation is affected by two factors. On the one hand, at high Cr-deficient level, a CuO phase appears. The mixed valency of Cu^+^/Cu^2+^ results in an enhancement of the hybridization between Cu*3d* and O*2p* orbitals, which reduces the localization of the holes by oxygen and leads to a high carrier mobility [30]. On the other hand, the appearance of CuO impurity phase enhances the grain boundary scattering and thereby reduces the carrier mobility. In this work, the carrier mobility reduced from 0.84 to 0.04 cm^2^·V^−1^·s^−1^ as Cr content decreased from 0.95 to 0.58, indicating the grain boundary scattering plays a dominant role in the carrier mobility variation.

**Figure 3:**
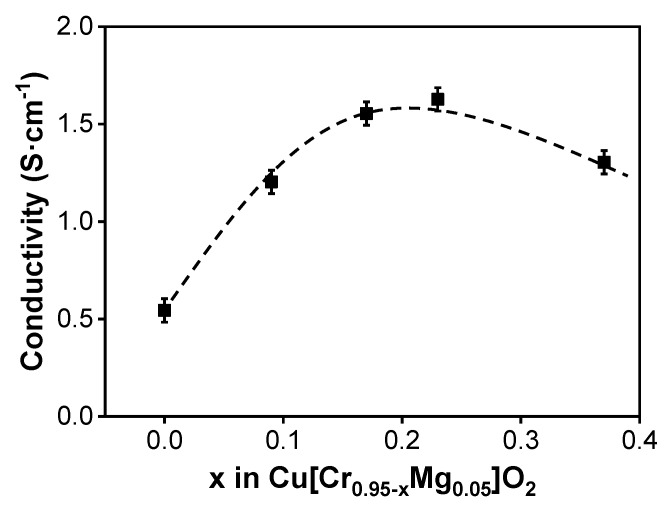
The electrical conductivity of Cr-deficient Cu[Cr_0.95−x_Mg_0.05_]O_2_ films as a function of Cr content.

The electrical conductivity of Cr-deficient Cu[Cr_0.95−x_Mg_0.05_]O_2_ films as a function of Cr content is shown in Figure 3. Under the combined effect of carrier concentration and carrier mobility, the film’s conductivity firstly increased and then decreased. This implies that, under high Cr-deficient level, the negative influence of the grain boundary scattering from CuO impurity phase exceeded the contribution of Cu^2+^ to the carrier concentration. Then, among the five samples prepared in the current work, the optimal p-type conductivity of about 1.63 S·cm^−1^ is achieved in Cu[Cr_0.72_Mg_0.05_]O_2_ film, where the carrier concentration and carrier mobility are 1.3 × 10^20^ cm^−3^ and 0.08 cm^2^·V^−1^·s^−1^, respectively.

**Figure 4:**
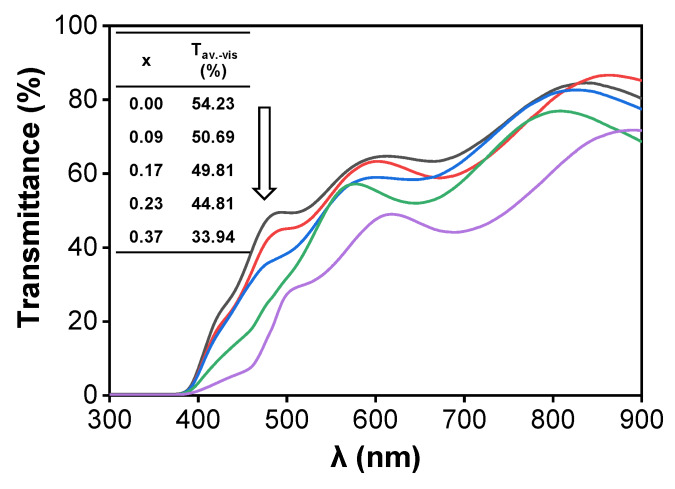
The transmittance in the visible region of Cr-deficient Cu[Cr_0.95−x_Mg_0.05_]O_2_ films as a function of Cr content.

The film’s transmittance variation in the visible region (400–800 nm) is shown in Figure 4. The average transmittance in the visible region was obtained with the following equation [31]:

(3) $$T_{average}=\frac{\int_{\lambda_{1}}^{\lambda_{n}}T\left(\lambda \right)d\lambda }{\lambda_{n}-\lambda_{1}}\approx \frac{1}{m}\overset{m}{\underset{\lambda =\lambda_{1}}{\sum }}T\left(\lambda \right)\;\left(m=\lambda_{1},\lambda_{2},\lambda_{3}\ldots \lambda_{n}\right)$$

where *λ_1_* = 400 nm and *λ_n_* = 800 nm. It clearly shows that the film’s transmittance reduces when Cr- deficiency increases. This phenomenon may be caused by the variation of the film’s crystallinity and the appearance of a CuO impurity phase. More light scattering is introduced into the film, thus reducing the film’s transmittance. As for the stoichiometric Cu[Cr_0.95_Mg_0.05_]O_2_ film, its average transmittance in the visible region amounts to 54.23%.

**Figure 5:**
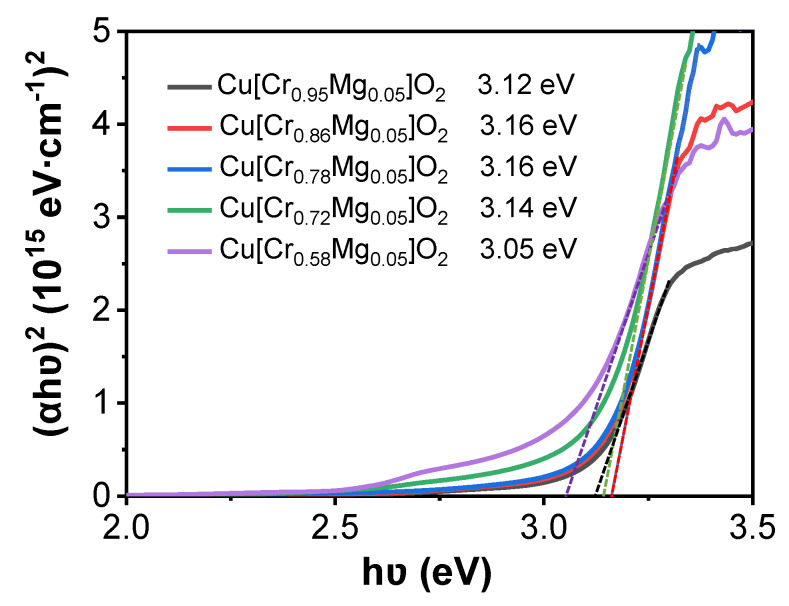
The variation of direct band gap of Cu[Cr_0.95-x_Mg_0.05_]O_2_ films.

**Figure 6:**
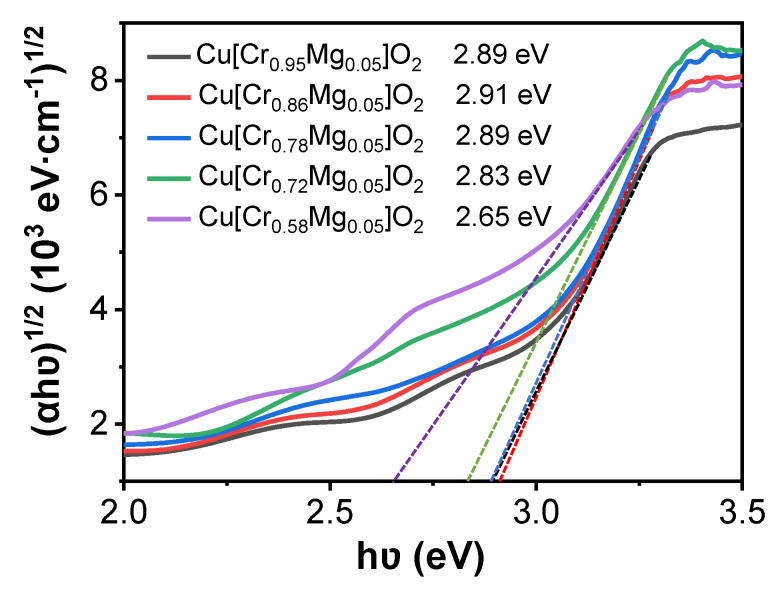
The variation of indirect band gap of Cu[Cr_0.95-x_Mg_0.05_]O_2_ films.

The optical band gap *E_g_* of Cu[Cr_0.95−x_Mg_0.05_]O_2_ can be estimated by the following formulas [32,33]:

(4) $$\alpha =\frac{1}{d}\ln\left(\frac{1-R}{T}\right)$$

(5) $${\left(\alpha hv\right)}^{1/n}=A\left(hv-Eg\right)$$

where *d* is film thickness, *R* and *T* are optical reflectance and transmittance, respectively. *hυ* is the incident photon energy, *A* is a constant and the exponent *n* depends on the type of transition: *n* = 1/2 and 2 for direct and indirect transition, respectively. Since CuCrO_2_ has a direct band gap transition, *n* equals 1/2 in this equation. Figure 5 depicts the variation of the direct band gap ($E_{g}^{d}$) of Cu[Cr_0.95−x_Mg_0.05_]O_2_ film. As Cr content decreased from 0.95 to 0.86, 0.78, 0.72 and 0.58, the film’s corresponding direct band gap varied from 3.12 to 3.16, 3.16, 3.14, and 3.05 eV. At first, the increment in the film’s band gap was primarily caused by the Burstein–Moss effect, which is often found in degenerate semiconductors [31]. In the present work, as Cr deficiency increased, the Cu[Cr_0.95−x_Mg_0.05_]O_2_ films became degenerate as the carrier concentration was higher than 10^19^ cm^−3^ [29]. Meanwhile, the Fermi level of Cu[Cr_0.95−x_Mg_0.05_]O_2_ moved towards the valence band. In this condition, only electrons below the Fermi level can be excited to the conduction band because no states above the Fermi level are filled with electrons. As a result, the Burstein–Moss effect led to a greater band gap. This behavior is more pronounced in the Cr-deficient film, where the carrier concentration was higher than others. Thus, the film’s band gap became wider with increasing Cr deficiency. Similar behavior has also been reported in other works [34,35]. However, as Cr deficiency further increased, the film’s band gap narrowed when a CuO phase appears. This was mainly caused by the narrower band gap of CuO of about 1.25 eV greatly enhancing light absorption [36]. As a result, the film’s band gap reduced. Considering the debate over the nature of the band gap of CuCrO_2_ [37], the indirect band gaps of the films were also estimated, and the estimates are shown in Figure 6. Its variation is the same as that of the direct band gap. Benko et al. found that an indirect allowed transition at 3.08 eV exists in CuCrO_2_ [38]. Rastogi et al. also reported that CuCrO_2_ film with a thickness of 305 nm possesses an indirect band gap of 2.79 eV [39].

**Figure 7:**
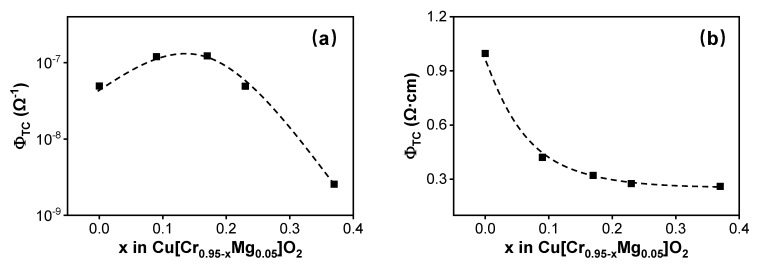
Figure of merit (FOM) defined as (**a**) Haacke’s figure of merit, (**b**) customized in this work of Cu[Cr_0.95−x_Mg_0.05_]O_2_ films with various Cr content.

Finally, Haacke’s figure of merit (FOM, *Φ_TC_*) was used to evaluate the quality of the films [40]. It is defined as

(6) $$\Phi_{TC}=\frac{T^{10}}{R_{sh}}$$

where *T* is the average transmittance in the visible region and *R_sh_* is the sheet resistance. The *Φ_TC_* values obtained for different films are compared in Figure 7a. Under the influence of the electrical and optical properties, *Φ_TC_* value first increased and then reduced. The optimal value was realized when the film’s composition was Cu[Cr_0.78_Mg_0.05_]O_2_, where the conductivity and the average transmittance in the visible region were 1.55 S·cm^−1^ and 49.81%, respectively.

Haacke’s figure of merit has been widely used to evaluate n-type TCO materials, but for p-type TCO, whether it is still preferred is controversial. As a result, another figure of merit defined as shown in Equation (7) was also selected here. Its variation with the film’s composition is shown in Figure 7b.

(7) $$\Phi_{TC}=\frac{T}{\sigma }$$

where *σ* is the film’s conductivity. It can be seen that in this definition of figure of merit, the film’s transmittance plays a dominant role when assessing the film’s optoelectronic properties. The value of this figure of merit reduces as Cr content decreases.

## 4. Conclusions

Mg doped Cr-deficient Cu[Cr_0.95−x_Mg_0.05_]O_2_ films were deposited by the reactive magnetron sputtering in this work. The influence of Cr deficiency on the film’s optoelectronic properties was investigated. Thanks to the conversion of monovalent copper to divalent copper with an increase in Cr deficiency, the hybridization between Cu*3d* and O*2p* orbitals was enhanced, which was beneficial in reducing the localization of holes by oxygen. As a result, the carrier concentration increased. However, as Cr content was as low as 0.58 in Cu[Cr_0.58_Mg_0.05_]O_2_, the secondary phase of CuO was observed. This impurity phase introduced more grain boundary scattering and impeded the transmission of carriers and photon. Therefore, the carrier mobility as well as the film’s transmittance reduced with increased Cr deficiency. In the current work, if the film’s optoelectronic performance is evaluated by Haacke’s figure of merit, the optimal one is achieved as Cr content is 0.78 in Cu[Cr_0.78_Mg_0.05_]O_2_. Its Haacke’s figure of merit is about 1.23 × 10^−7^ Ω^−1^.
